# Data on major power outage events in the continental U.S.

**DOI:** 10.1016/j.dib.2018.06.067

**Published:** 2018-06-26

**Authors:** Sayanti Mukherjee, Roshanak Nateghi, Makarand Hastak

**Affiliations:** a Lyles School of Civil Engineering, Division of Construction Engineering and Management, and School of Industrial Engineering, Purdue University, West Lafayette, IN 47907, USA; b School of Industrial Engineering and Division of Environmental and Ecological Engineering, Purdue University, West Lafayette, IN 47907, USA; c Lyles School of Civil Engineering and Division of Construction Engineering and Management, Purdue University, West Lafayette, IN 47907, USA

## Abstract

This paper presents the data that is used in the article entitled “A Multi-Hazard Approach to Assess Severe Weather-Induced Major Power Outage Risks in the U.S.” (Mukherjee et al., 2018) [1]. The data described in this article pertains to the major outages witnessed by different states in the continental U.S. during January 2000–July 2016. As defined by the Department of Energy, the major outages refer to those that impacted atleast 50,000 customers or caused an unplanned firm load loss of atleast 300 MW. Besides major outage data, this article also presents data on geographical location of the outages, date and time of the outages, regional climatic information, land-use characteristics, electricity consumption patterns and economic characteristics of the states affected by the outages. This dataset can be used to identify and analyze the historical trends and patterns of the major outages and identify and assess the risk predictors associated with sustained power outages in the continental U.S. as described in Mukherjee et al. [1].

## Specifications Table

**Table:** 

Subject area	*Risk and reliability*
More specific subject area	*Major power outages, Severe weather-induced outages, Natural hazards, Electricity service reliability*
Type of data	*Table, Excel file*
How data was acquired	*Using different publicly available datasets such as: (i) OE-417 form Schedule 1 published by DOE׳s Office of Electricity Delivery and Energy Reliability*[2]*(ii) U.S. Energy Information Administration (EIA) [form EIA-826 and EIA-861]*[3]*; (iii) National Oceanic and Atmospheric Administration (NOAA) and National Climatic Data Center (NCDC)*[4]*; (iv) U.S. Department of Labor; Bureau of Labor Statistics*[5]*; (v) U.S. Census Bureau.*
Data format	*Raw; Aggregated, Filtered*
Experimental factors	*Not applicable*
Experimental features	*Statistical analysis of the data leveraging a hybrid classification-regression model to identify and estimate the influence of various predictors attributing to increased risk of sustained power outages*
Data source location	*All the states in the continental U.S.*
Data accessibility	*Data is available within this article in the link provided*

## Value of the data

• This dataset serves as a rich repository of various information related to the major outage patterns, and characteristics of the states in the continental U.S., including their climate and topographical characteristics, electricity consumption patterns, population, and land-cover characteristics.
• This data provides valuable information that can be used to conduct future research in various paradigms, such as—state-level power outage risk maps for the continental U.S., predicting demand load loss, analyzing vulnerability of the U.S. states to frequent major power outages, and studying historical trends of major power outages.
• The aggregated and filtered data would also help the researchers to test various types of hypothesis of their interest in the future, especially in the areas of utility planning, risk management, and policy analysis.
• This dataset can be also leveraged to replicate the results corresponding to the original article following the data preparation procedures and the methodology as proposed in [1].

## 1 Data

The data presented in this article is included in a single excel file containing 55 variables. The excel file can be accessed from the link: https://engineering.purdue.edu/LASCI/research-data/outages/outagerisks. The variable measures are given in Imperial System of Measurement. The variable descriptions are summarized in Table 1. This data contains valuable information related to the severe weather-induced major power outages and the various regional characteristics that might attribute to the growing risks of such outages.

**Table 1:** Variable descriptions.

**Variable types**	**Variable names**	**Description**
**GENERAL INFORMATION**		
**Time of the outage event**	YEAR	Indicates the year when the outage event occurred
	MONTH	Indicates the month when the outage event occurred
**Geographic areas**	U.S._STATE	Represents all the states in the continental U.S.
	POSTAL.CODE	Represents the postal code of the U.S. states
	NERC.REGION	The North American Electric Reliability Corporation (NERC) regions involved in the outage event
**REGIONAL CLIMATE INFORMATION**		
**U.S. Climate regions**	CLIMATE.REGION	U.S. Climate regions as specified by National Centers for Environmental Information (nine climatically consistent regions in continental U.S.A.)
**El Niño/La Niña**	ANOMALY.LEVEL	This represents the oceanic El Niño/La Niña (ONI) index referring to the cold and warm episodes by season. It is estimated as a 3-month running mean of ERSST.v4 SST anomalies in the Niño 3.4 region (5°N to 5°S, 120–170°W) [6]
	CLIMATE.CATEGORY	This represents the climate episodes corresponding to the years. The categories—“Warm”, “Cold” or “Normal” episodes of the climate are based on a threshold of ± 0.5 °C for the Oceanic Niño Index (ONI)
**OUTAGE EVENTS INFORMATION**		
**Event start and end information**	OUTAGE.START.DATE	This variable indicates the day of the year when the outage event started (as reported by the corresponding Utility in the region)
	OUTAGE.START.TIME	This variable indicates the time of the day when the outage event started (as reported by the corresponding Utility in the region)
	OUTAGE.RESTORATION.DATE	This variable indicates the day of the year when power was restored to all the customers (as reported by the corresponding Utility in the region)
	OUTAGE.RESTORATION.TIME	This variable indicates the time of the day when power was restored to all the customers (as reported by the corresponding Utility in the region)
**Cause of the event**	CAUSE.CATEGORY	Categories of all the events causing the major power outages
	CAUSE.CATEGORY.DETAIL	Detailed description of the event categories causing the major power outages
	HURRICANE.NAMES	If the outage is due to a hurricane, then the hurricane name is given by this variable
**Extent of outages**	OUTAGE.DURATION	Duration of outage events (in minutes)
	DEMAND.LOSS.MW	Amount of peak demand lost during an outage event (in Megawatt) [but in many cases, total demand is reported]
	CUSTOMERS.AFFECTED	Number of customers affected by the power outage event
**REGIONAL ELECTRICITY CONSUMPTION INFORMATION**		
**Electricity price**	RES.PRICE	Monthly electricity price in the residential sector (cents/kilowatt-hour)
	COM.PRICE	Monthly electricity price in the commercial sector (cents/kilowatt-hour)
	IND.PRICE	Monthly electricity price in the industrial sector (cents/kilowatt-hour)
	TOTAL.PRICE	Average monthly electricity price in the U.S. state (cents/kilowatt-hour)
**Electricity consumption**	RES.SALES	Electricity consumption in the residential sector (megawatt-hour)
	COM.SALES	Electricity consumption in the commercial sector (megawatt-hour)
	IND.SALES	Electricity consumption in the industrial sector (megawatt-hour)
	TOTAL.SALES	Total electricity consumption in the U.S. state (megawatt-hour)
	RES.PERCEN	Percentage of residential electricity consumption compared to the total electricity consumption in the state (in %)
	COM.PERCEN	Percentage of commercial electricity consumption compared to the total electricity consumption in the state (in %)
	IND.PERCEN	Percentage of industrial electricity consumption compared to the total electricity consumption in the state (in %)
**Customers served**	RES.CUSTOMERS	Annual number of customers served in the residential electricity sector of the U.S. state
	COM.CUSTOMERS	Annual number of customers served in the commercial electricity sector of the U.S. state
	IND.CUSTOMERS	Annual number of customers served in the industrial electricity sector of the U.S. state
	TOTAL.CUSTOMERS	Annual number of total customers served in the U.S. state
	RES.CUST.PCT	Percent of residential customers served in the U.S. state (in %)
	COM.CUST.PCT	Percent of commercial customers served in the U.S. state (in %)
	IND.CUST.PCT	Percent of industrial customers served in the U.S. state (in %)
**REGIONAL ECONOMIC CHARACTERISTICS**		
**Economic outputs**	PC.REALGSP.STATE	Per capita real gross state product (GSP) in the U.S. state (measured in 2009 chained U.S. dollars)
	PC.REALGSP.USA	Per capita real GSP in the U.S. (measured in 2009 chained U.S. dollars)
	PC.REALGSP.REL	Relative per capita real GSP as compared to the total per capita real GDP of the U.S. (expressed as fraction of per capita State real GDP & per capita US real GDP)
	PC.REALGSP.CHANGE	Percentage change of per capita real GSP from the previous year (in %)
	UTIL.REALGSP	Real GSP contributed by Utility industry (measured in 2009 chained U.S. dollars)
	TOTAL.REALGSP	Real GSP contributed by all industries (total) (measured in 2009 chained U.S. dollars)
	UTIL.CONTRI	Utility industry׳s contribution to the total GSP in the State (expressed as percent of the total real GDP that is contributed by the Utility industry) (in %)
	PI.UTIL.OFUSA	State utility sector׳s income (earnings) as a percentage of the total earnings of the U.S. utility sector׳s income (in %)
**REGIONAL LAND-USE CHARACTERICS**		
**Population**	POPULATION	Population in the U.S. state in a year
	POPPCT_URBAN	Percentage of the total population of the U.S. state represented by the urban population (in %)
	POPPCT_UC	Percentage of the total population of the U.S. state represented by the population of the urban clusters (in %)
	POPDEN_URBAN	Population density of the urban areas (persons per square mile)
	POPDEN_UC	Population density of the urban clusters (persons per square mile)
	POPDEN_RURAL	Population density of the rural areas (persons per square mile)
**Land area**	AREAPCT_URBAN	Percentage of the land area of the U.S. state represented by the land area of the urban areas (in %)
	AREAPCT_UC	Percentage of the land area of the U.S. state represented by the land area of the urban clusters (in %)
	PCT_LAND	Percentage of land area in the U.S. state as compared to the overall land area in the continental U.S. (in %)
	PCT_WATER_TOT	Percentage of water area in the U.S. state as compared to the overall water area in the continental U.S. (in %)
	PCT_WATER_INLAND	Percentage of inland water area in the U.S. state as compared to the overall inland water area in the continental U.S. (in %)

Note: “NA” in the data file indicates that data was not available.

## 2 Experimental design, materials and methods

The data on major power outages and the characteristics of the regions witnessing the outages were obtained from various publicly available data sources such as the: (i) OE-417 form Schedule 1 published by DOE׳s Office of Electricity Delivery and Energy Reliability [2] (ii) U.S. Energy Information Administration (EIA) [form EIA-826 and EIA-861] [3]; (iii) National Oceanic and Atmospheric Administration (NOAA); (iv) National Climatic Data Center (NCDC); (v) U.S. Department of Labor; Bureau of Labor Statistics [5]; and, (vi) U.S. Census Bureau. The data spans from January 2000 to July 2016. The various data sources were then aggregated using the year, month and the region (i.e., the U.S. state) as the nexus. The major outages are described in terms of duration of the outage event and the total number of customers affected during that event. The dataset is rigorously preprocessed and checked for inconsistencies to minimize the measurement errors leveraging different methods such as data visualization, analyzing the descriptive statistics as well as manual cross-checking of the observations.
